# Isolation and *in vitro* evaluation of bacteriophages against MDR-bacterial isolates from septic wound infections

**DOI:** 10.1371/journal.pone.0179245

**Published:** 2017-07-18

**Authors:** Roja Rani Pallavali, Vijaya Lakshmi Degati, Dakshayani Lomada, Madhava C. Reddy, Vijaya Raghava Prasad Durbaka

**Affiliations:** 1 Department of Microbiology, Yogi Vemana University, Kadapa, AP, India; 2 Department of Genetic and Genomics, Yogi Vemana University, Kadapa, AP, India; 3 Department of Biotechnology and Bioinformatics, Yogi Vemana University, Kadapa, AP, India; Jawaharlal Nehru University, INDIA

## Abstract

Multi-drug resistance has become a major problem for the treatment of pathogenic bacterial infections. The use of bacteriophages is an attractive approach to overcome the problem of drug resistance in several pathogens that cause fatal diseases. Our study aimed to isolate multi drug resistant bacteria from patients with septic wounds and then isolate and apply bacteriophages *in vitro* as alternative therapeutic agents. Pus samples were aseptically collected from Rajiv Gandhi Institute of Medical Science (RIMS), Kadapa, A.P., and samples were analyzed by gram staining, evaluating morphological characteristics, and biochemical methods. MDR-bacterial strains were collected using the Kirby-Bauer disk diffusion method against a variety of antibiotics. Bacteriophages were collected and tested *in vitro* for lytic activity against MDR-bacterial isolates. Analysis of the pus swab samples revealed that the most of the isolates detected had *Pseudomonas aeruginosa* as the predominant bacterium, followed by *Staphylococcus aureus*, *Klebsiella pneumoniae* and *Escherichia coli*. Our results suggested that gram-negative bacteria were more predominant than gram-positive bacteria in septic wounds; most of these isolates were resistant to ampicillin, amoxicillin, penicillin, vancomycin and tetracycline. All the gram-positive isolates (100%) were multi-drug resistant, whereas 86% of the gram-negative isolates had a drug resistant nature. Further bacteriophages isolated from sewage demonstrated perfect lytic activity against the multi-drug resistant bacteria causing septic wounds. *In vitro* analysis of the isolated bacteriophages demonstrated perfect lysis against the corresponding MDR-bacteria, and these isolated phages may be promising as a first choice for prophylaxis against wound sepsis, Moreover, phage therapy does not enhance multi-drug resistance in bacteria and could work simultaneously on a wide variety of MDR-bacteria when used in a bacteriophage cocktail. Hence, our results suggest that these bacteriophages could be potential therapeutic options for treating septic wounds caused by *P*. *aeruginosa*, *S*. *aureus*, *K*. *pneumoniae* and *E*. *coli*.

## Introduction

The skin is the largest sensory organ, and it provides innate immunity and protects the underlying tissues of the human body. The most important function of the skin is to provide protection against pathogenic microbes, which invade the skin, and to control bacterial colonization [1]. The loss of skin integrity by any mechanical injuries exposes subcutaneous tissues to the environment, which leads to microbial colonization and proliferation [2]. Mechanical disruption of the skin results in a wound, and it is the major cause of the establishment of infections by microorganisms ranging from bacteria and fungi to parasites and viruses [3]. Septic infections are caused mostly by bacteria; they break the protection barrier [4,5] and may establish deep infections.

A septic wound is a type of infection that can have an antagonistic impact on the human body, quality of life and on the healing rate of the wound. Wound infections are reported in one third of hospital acquired infections among surgical patients and account for 70–80% of mortality. Wound infections are primary factors in the development of morbidity and mortality in patients, particularly in developing countries, irrespective of the nature of the wound [6,7]. Wounds usually provide adequate warmth, moisture and nutrition conditions for favorable growth and proliferation of microorganisms [8].

Bacterial infections in wound patients are common and difficult to control, particularly in the hospital environment. Septic wounds, which harbor multiple pathogenic bacteria, are common and lead to sepsis [9,10]. The diagnosis of wound infections is a serious problem requiring an inordinately long time, usually requiring sophisticated diagnostic equipment or qualified professionals [9,11].

There are different types of wound infections, such as surgical wound infections, acute soft tissue infections, bite wound infections, burn wound infections and pyogenic wound infections. Wounds may not easily subside, and they may spread because of human habits. They are also aggravated in patients with disorders like diabetes, obesity and cardiovascular diseases. Infectious wounds are critical, painful, odorous and hypersensitive and lead to discomfort and inconvenience for patients. A number of studies conducted recently revealed that for septic wounds, the most common isolates are *S*. *aureus*, *P*. *aeruginosa*, *E*. *coli*, *Klebsiella* spp. and *Acinetobacter* spp. [12]. The control of wound infections has become challenging due to the widespread bacterial resistance to antibiotics and greater incidence of infections caused by poly-microbial flora [13].

At present, a number of antibiotics currently in use are becoming ineffective to control bacterial pathogens because many bacteria have attained multi-drug resistance. The indiscriminate and wide spread use of antibiotics has caused drug resistance in bacteria because of the over administration, self-medication, random prescription of improper drugs and prolonged use of antibiotics [14]. Further, antibiotic resistance gene transfer by conjugation leads to the evolution of resistant microbes [15,16]. Antibiotics are wonder drugs; however, because of MDR-bacterial emergence, it is imperative to develop alternative methods to treat MDR-bacterial pathogens [17]. Antibiotic resistant "superbugs" have become one of the world's most important public health concerns. Many diseases are becoming increasingly resistant to commonly used antibiotics because of elevated numbers of antibiotic resistant “superbugs.” In Europe, drug resistant microorganisms cause 25,000 deaths per year, and in the United States, 23,000 deaths per year are connected to MDR-bacterial infections. WHO reports show that drug resistance in bacteria has been found in all regions of the world, accounting for approximately 50% of *E*. *coli*, *K*. *pneumoniae*, *S*. *aureus* and *P*. *aeruginosa* infections that were resistant to most potent antibiotics, such as cephalosporin, which is a third-generation drug resulting in a high mortality rate with these drug resistant bacterial isolates. In view of this, the WHO advisory committee advocated for a new method of therapeutic intervention instead of antibiotic treatment. Furthermore, clinicians and scientists working in this area have demonstrated renewed interest in phage therapy against MDR-bacteria [18].

Several studies have been initiated to standardize alternatives to antibiotics that utilize novel mechanisms of action to achieve antibacterial activity. These approaches include bacteriophage therapy, iron chelation therapy, antimicrobial peptides, prophylactic vaccination, photodynamic therapy, and nitric oxide (NO)-based therapies for the containment of pathogenic bacteria. However, before clinically applying these practices, a number of routine standardizations are required, and there are still limitations that need to be addressed in each of the mentioned methods.

A crucial problem with the use of antibiotics is the appearance of resistant bacteria, so currently, much attention is focused on the application of bacteriophages as therapeutic agents [19]. Interestingly, bacteriophage therapy was widely practiced in the Eastern world before antibiotic discovery, but it was never fully established in the Western world. The success of phage therapy depends on identifying strategies to cure infections and reduce the emergence of phage resistant bacteria [20,21].

Sewage and hospital waste are ready sources of bacteriophages; in addition, phage purification and production costs are much cheaper than those of antibiotics. Bacteriophages are viruses that infect bacteria, and they are obligate intracellular parasites replicating within the host using the enzymatic machinery of the host. Bacteriophages show extreme host specificity, infecting particular strains even among single bacterial species, although some bacteriophages may infect multiple species [22]. A number of *in vitro* studies have shown that bacteriophages have the potential to lyse targeted bacterial pathogens [23].

The present work was carried out to investigate the possibility of using a lytic phage to treat MDR-bacteria infecting septic wounds. The study was designed to determine the prevalence of MDR-bacteria in isolates from septic wounds, to isolate and evaluate the *in vitro* efficacy of bacteriophages against MDR-bacterial isolates and to establish an alternative strategy to antibiotics for managing wound infections causing multi-drug resistant bacteria in hospital environment.

## Materials and methods

### Sample collection

A total of 130 septic wound samples were collected from Rajiv Gandhi Institute of Medical sciences (RIMS), Kadapa, Andhra Pradesh, India, and within one hour, the collected samples were aseptically transported. The samples were segregated based on disease types. The samples were from 49 diabetic wounds, 69 post-operative wounds and 12 burn wounds. EMB agar was purchased from Qualigens, Mumbai, India. Syringe driven filters (0.45 μM), Luria-Bertani broth and agar, and the antibiotics amoxicillin, ampicillin, benzyl penicillin, streptomycin, tetracycline, tobramycin, vancomycin, kanamycin, ciprofloxacin, cefotaxime and gentamycin were obtained from Hi-Media Laboratories, Mumbai, India.

### Culture and identification

Collected samples were inoculated on selective media (Mannitol salt agar, MacConkey agar, EMB agar, Blood agar and Cetrimide agar), and after the appropriate incubation period, the cultures were analyzed for morphological, physiological and biochemical characteristics. Biochemical tests included catalase activity, oxidase test, IMVIC test, carbohydrate fermentation test, mannitol fermentation test, nitrate reduction, urease production and coagulase test.

### Antibiotic susceptibility test (AST)

The predominant bacterial isolates from septic wound patients were tested for antibiotic susceptibility patterns with 11 different antibiotics belonging to six classes. The antibiotics employed for the study were benzyl penicillin, amoxicillin, ampicillin, kanamycin, tobramycin, gentamycin streptomycin, cefotaxime, vancomycin, tetracycline and ciprofloxacin. Antimicrobial susceptibility patterns were detected with the standard protocol [24] Kirby-Bauer disk diffusion method on Mueller-Hinton agar. After the incubation period, the diameters of the zone of inhibition around the discs were measured using a ruler and then classified as sensitive, intermediate, or resistant, according to the standardized table supplied by CLSI guidelines [25].

### Isolation of bacteriophages

To isolate lytic bacteriophages against the predominant MDR-bacteria (MDR *S*. *aureus*, *P*. *aeruginosa*, *K*. *pneumoniae and E*. *coli*), colonies were selected and collected from the colonies from nutrients and the selective media agar plates for further study (S1 Fig). Bacteriophages against these MDR-strains were collected from raw, stagnant sewage water of the municipal sewage plant at RIMS using the method of Smith and Huggins [26]. Then, the Cerveny method was used to enrich bacteriophages [27] with small modifications. The collected sewage samples were centrifuged at 5000 g for 10 minutes, and then, the supernatant was collected and filtered through 0.45 μM syringe driven filters (Hi-Media, Mumbai). After that, approximately 0.5 mL of chloroform was added to 50 mL of the filtrate and then incubated for 20 minutes [28,29], and then, 5 mL of the corresponding bacterial cultures (in early log phase, OD at 0.4–0.7) and 20 mL of 2X LB broth were added and then incubated overnight at 37°C. After 24 h, the broth cultures demonstrating visible lysis were pelleted by centrifugation at 4°C at 8000 g for 20 min and filtered through 0.45 *μ*M syringe driven filters. Chloroform does not affect the activity of bacteriophages; moreover, it kills bacteria present in the phage filtrates [28,29]. Bacterial cell debris was removed by filtration, and the obtained filtrates were tested for lytic phages using Adams double layer agar method with small modifications (5% glycerol) [30] and spot assays [31]. The titers of each phage, isolated against MDR-bacteria from septic wounds, were expressed in plaque forming units (PFU^-1^), and the antibacterial efficacy of isolated phages was evaluated by a spot assay and by the double layer agar method, as described by Sambrook [32].

### Double layer agar method

200 μL of bacterial culture (in log phase 10^9^ CFU^-1^) and 100 μL of purified phage filtrates (10^9^ PFU^-1^) were mixed and incubated for 5 min for proper adsorption. Later, these bacteria-phage filtrates were placed in a sterile tube mixed with 5 mL soft agar (0.7% agar) and poured onto the bottom agar and then swirled to produce a uniform top layer. The plates were incubated at 37°C for 24 h for plaque formation. After incubation, the formation of cleared zones (plaques) suggested the presence of lytic phages. The plaques were collected and resuspended in salt of magnesium (SM) buffer (100 mM Nacl, 8 mM MgSO_4_, 50 mM Tris-Cl (pH 7.5), and 0.01% gelatin), and then, these phage lysates were used to test the lytic efficacy of bacteriophages *in vitro*.

#### Spot assay

The spot assay was used to screen the bactericidal ability of the isolated phages [31]. Bacterial isolates from septic wounds were grown in LB broth. After the cultures reached the early log phase (OD at 600 nm, 0.5–0.7), 200 μL of bacteria was mixed with 5 mL soft agar and poured onto the bottom agar to solidify. Then, 10 μL of phage filtrate was collected and spotted on the soft agar at a titer of 10^9^ PFU^-1^ on LB agar. The plates were allowed to dry and examined for lysis zones or plaque formation after overnight incubation at 37°C.

### *In vitro* phage therapy

One well-formed plaque containing bacteriophages from each agar plate was selected for the *in vitro* assay. The plaques from agar plates were diluted in SM buffer, and then, chloroform (1%) was added to remove bacteria. The phage lysates were centrifuged, and the supernatant was used to test lytic activity against MDR-bacterial strains from septic wounds. MDR-strains of *P*. *aeruginosa*, *S*. *aureus*, *K*. *pneumoniae* and *E*. *coli* (1 mL) were separately inoculated in two flasks with 150 mL LB media. To evaluate the *in vitro* lytic efficacy of bacteriophages, 500 μL phage filtrates of multiplicity of infection (m.o.i) 1 and 10 were transferred into test flasks containing 150 mL LB broth with MDR-bacteria. In parallel, controls were set up with flasks containing the respective MDR-bacteria alone. The test and control flasks were incubated in a shaking incubator at 37°C at 120 g for 24 h. The OD 600 values were recorded after every 2 h for a time period of 24 h using a UV-spectrophotometer, and the obtained values at m.o.i (multiplicity of infection) 1 and 10 values were compared with controls. Duplicates were maintained for each set to analyze the results.

#### Statistical analysis

Differences in sex, the number of infected and uninfected patients, age groups and the number of infected and uninfected patients, and wound types with gram-positive and gram-negative bacterial isolates were analyzed using Chi-squared tests. A difference with P˂0.05 was considered statistically significant.

### Ethics

This experimental study was approved by the **Institutional Ethics Committee for Human Research** (IECHR), and all procedures were conducted in accordance with the “Guide for the Care and Use of Laboratory.”

## Results

From a pool of the 130 samples collected and tested, 102 (78.4%) swab samples showed microbial growth, and no appropriate growth was noticed in the remaining 28 samples (21.5%). Forty-nine (37.6%) samples screened were obtained from subjects suffering from diabetic wounds, while 12 (9.2%) of the samples were from burn patients and 69 samples (53%) were from post-operative wounds. The percentages of samples collected in the selected hospital for the study indicated that there were significantly more patients with post-operative wounds than patients with diabetic or burn wounds. The outcome of the study clearly indicated that the number of infections in males (69%) was higher than that in females (31%). These findings were recorded for the age group consisting of subjects between 8–80 years. Wound infection is common in old patients [33].

### Bacterial profile

**Fig 1:**
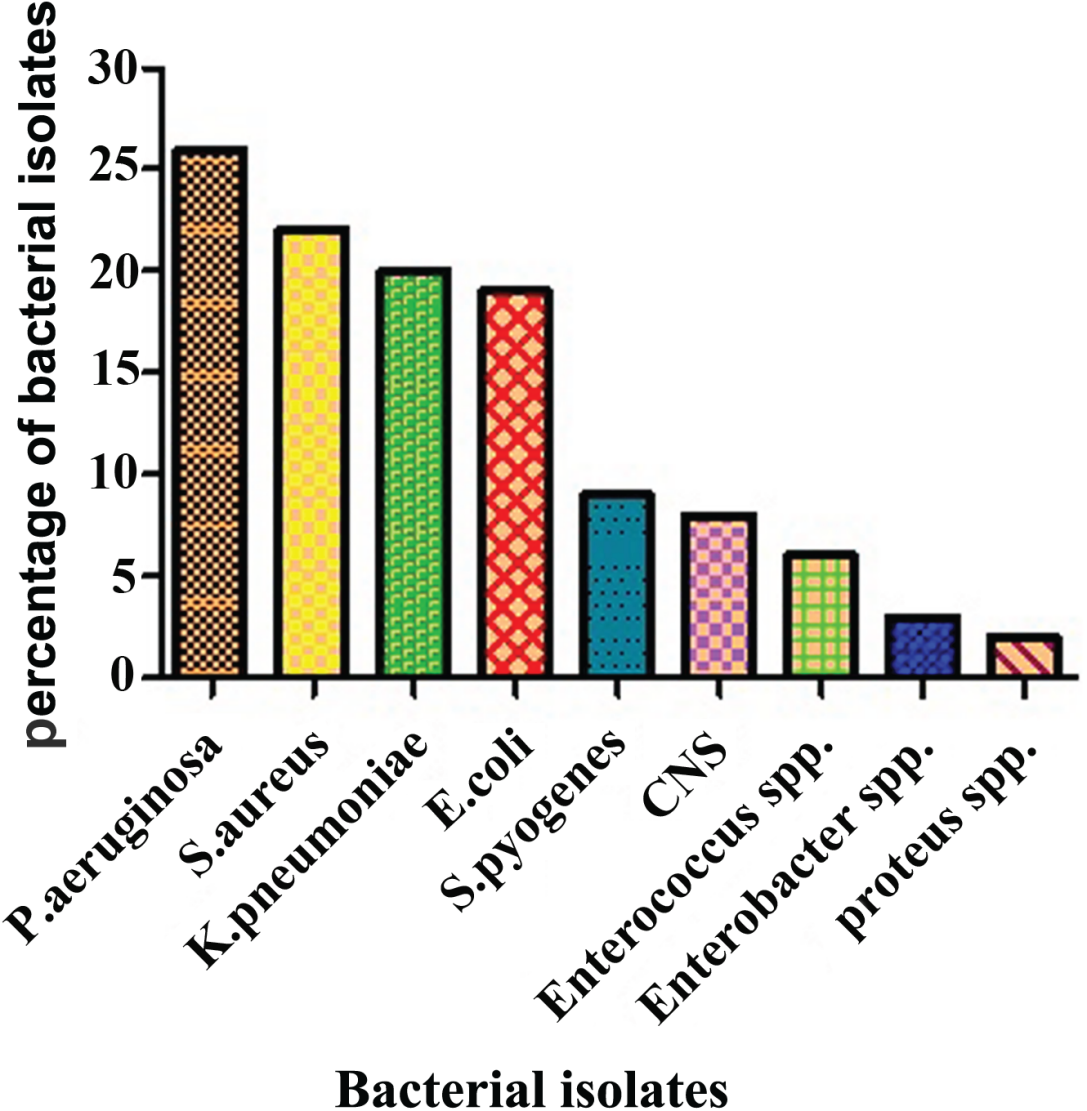
Predominant bacterial isolates from septic wound patients.

Among 130 swabs, 102 (78.4%) were positive for bacterial pathogens, while 28 (21.5%) were bacteriologically sterile. Most frequently, only a single species was isolated from each sample, accounting for 95 samples(93.1%), while more than one species was isolated from 7 (6.8%) of the total positive samples. A total of 115 bacterial isolates were obtained, 70 (60.8%) of which were gram-negative, while 45 (39.1%) were gram-positive. *Pseudomonas aeruginosa* was the most predominant bacteria isolated in 26 (22.6%) samples, followed by *Staphylococcus aureus* in 22 (19.1%), *Klebsiella pneumoniae* in 20 (17.3%), *Escherichia coli* (E. coli) in 19 (16.5%), *Streptococcus pyogenes* in 9 (7.8%), C*oagulase-negative Staphylococci* spp. 8 (6.9%), *Enterococcus* spp. 6 (5.2%), *Enterobacter* spp. 3 (2.6%) and *Proteus* spp. 2 (1.7%) (**Fig 1**).

The findings indicate that there was no significant association between sex and infection rate (P = 0.5447) at P > 0.05 (**Table 1**). There was a greater incidence of wound infection in the 60 years and older age group, so there is a significant association between age and incidence of wound infection (P = 0.0485) at P <0.05 (**Table 2**), and there is no statistical significance between the type of wound and the type of microorganisms isolated (P = 0.3072) at P > 0.05 (**Table 3**).

**Table 1:** Sex distribution of bacterial isolates from septic wounds.

Sex	Infected No. (%)	Un-Infected No. (%)	Total No. (%)	Chi- square test	P-Valve
**Male**	73 (81.1%)	17 (18.8%)	90 (69.2%)		
**Female**	29 (72.5%)	11 (27.5%)	40 (30.7%)	1.505	P = 0.82
**Total**	102 (78.4%)	28 (21.5%)	130 (100%)		

P- Value was calculated by using Graph pad Prism software.

**Table 2:** Age distribution of patients with significant bacterial growth.

Age group	Infected No. (%)	Un-Infected No. (%)	Total No. (%)	P-Valve
**0–10**	3 (100%)	0 (0%)	3 (2.3%)	
**11–20**	8 (72.7%)	3 (27.2%)	11 (8.4%)	
**21–30**	7 (53.8%)	6 (46.1%)	13 (10%)	
**31–40**	12 (63.1%)	7 (36.8%)	19 (14.6%)	P = 0.0485
**41–50**	15 (60%)	10 (40%)	25 (19.2%)	
**51–60**	24 (92.3%)	2 (7.6%)	26 (20%)	
**61 and above**	33 (100%)	0 (0%)	33 (25.3%)	
**Total**	102 (78.4%)	28 (21.5%)	130 (100%)	

P- Value was calculated by using Graph pad Prism software.

**Table 3:** Wound type with significant bacterial type.

Wound type	Gram positive bacteria		Gram negative bacteria		Total		P-Value
	**No**	**%**	**No**	**%**	**No**	**%**	
**Diabetic wounds N = 49**	10	28.5%	25	71.4%	35	30.4%	
**Burn wounds N = 12**	6	42.8%	8	57.1%	14	12.1%	P = 0.3072
**Post-operative wounds N = 69**	29	43.9%	37	56%	66	57.3%	
**Total**	45	100%	70	100%	115	100%	

P- Value was calculated by using Graph pad Prism software.

### Antibiotic susceptibility pattern of bacterial isolates

The most frequently encountered bacterial cultures were tested against the selected 11 antibiotic drugs belonging to six different classes of antibiotics based on the bacterial spectrum, route of administration and type of activity to isolate multi-drug resistant strains among the pool of samples. The drugs tested for both gram-negative and gram-positive bacteria were gentamycin (10 μg), streptomycin (20 μg), tetracycline (20 μg), vancomycin (20 μg), kanamycin (20 μg), benzyl penicillin (30 μg), ampicillin (40 μg), amoxicillin (30 μg), cefotaxime (30 μg), ciprofloxacin (10 μg) and tobramycin (20 μg). These antimicrobials were selected based on the availability and prescription frequency of these drugs in the study area.

**Fig 2:**
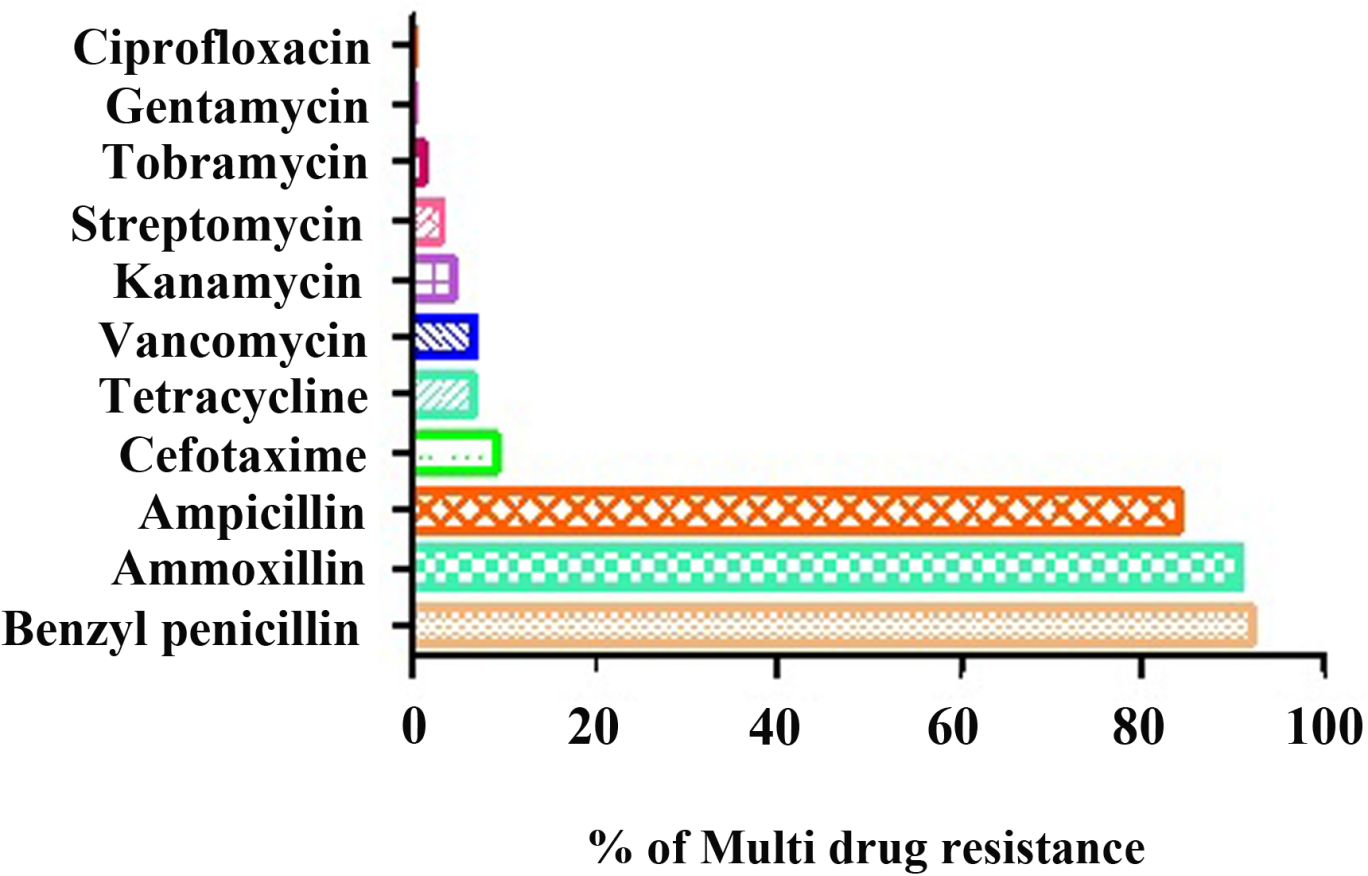
Percentage of multi-drug resistance against a variety of antibiotics in septic wound causing bacteria.

The results were obtained, as shown in the Table 4, and we observed that the organisms varied in their susceptibility to all the antimicrobial drugs used. Most demonstrated multiple resistance ability (resistance to two or more classes of antimicrobials) (**Fig 2**), and the antibiotic resistance patterns of isolates towards antibiotics are shown in Fig 2.

**Table 4:** Antibiotic susceptibility pattern of predominant bacterial isolates from septic wounds at RIMS, Kadapa.

Bacterial isolates	R/S Pattern						Antibiotics					
		**BP**	**AM**	**AP**	**KM**	**TM**	**SM**	**GM**	**T**	**VM**	**CF**	**CT**
***S*.*aureus***	**S**	0 (0.0%)	0 (0.0%)	0 (0.0%)	19 (86.3%)	21 (95.4%)	22 (100%)	22 (100%)	22 (100%)	20 (90.9%)	22 (100%)	20 (90.9%)
**N = 22**	**R**	22 (100%)	22 (100%)	22 (100%)	3 (13.6%)	1 (4.7%)	0 (0.0%)	0 (0.0%)	0 (0.0%)	2 (9.0%)	0 (0.0%)	2 (9.0%)
***P*.*aeruginosa***	**S**	2 (7.6%)	3 (11.5%)	5 (19.3%)	23 (88.4%)	26 (100%)	26 (100%)	26 (100%)	23 (88.4%)	23 (88.4%)	26 (100%)	16 (61.5%)
**N = 26**	**R**	24 (92.3%)	23 (88.4%)	21 (80.7%)	3 (11.3%)	0 (0.0%)	0 (0.0%)	0 (0.0%)	3 (11.3%)	3 (11.3%)	0 (0.0%)	10 (38.5%)
***K*.*pneumoniae***	**S**	3 (15%)	3 (15%)	5 (25%)	18 (90%)	20 (100%)	20 (100%)	20 (100%)	20 (100%)	19 (95%)	20 (100%)	16 (80%)
**N = 20**	**R**	17 (85%)	17 (85%)	15 (75%)	2 (10.0%)	0 (0.0%)	0 (0.0%)	0 (0.0%)	0 (0.0%)	1 (5%)	0 (0.0%)	4 (20%)
***E*.*coli***	**S**	2 (10.5%)	2 (10.5%)	3 (15.7%)	19 (100%)	19 (100%)	11 (57.8%)	19 (100%)	8 (42.1%)	19 (100%)	19 (100%)	19 (100%)
**N = 19**	**R**	17 (89.4%)	17 (89.4%)	16 (84.2%)	0 (0.0%)	0 (0.0%)	8 (42.1%)	0 (0.0%)	11 (57.8%)	0 (0.0%)	0 (0.0%)	0 (0.0%)
**Total**	**S**	7 (8.1%)	8 (9.1%)	13 (15%)	83 (95.4%)	86 (98.8%)	84 (96.5%)	87 (100%)	81 (93.1%)	81 (93.1%)	87 (100%)	79 (90.8%)
**N = 87**	**R**	80 (91.9%)	79 (90.8%)	74 (85%)	4 (4.5%)	1 (1.1%)	3 (3.4%)	0 (0.0%)	6 (6.8%)	6 (6.8%)	0 (0.0%)	8 (9.1%)

S- Sensitive, R- Resistant, BP-benzyl penicillin, AM-ammoxillin, AP-ampicillin, KM-kanamycin, TM-tobramycin, GM-gentamycin, SM- streptomycin, CT-cefotaxime, VM-vancomycin, T-tetracycline, CF-ciprofloxacin.

Antibiotic resistance to benzyl penicillin (91.9%), amoxicillin (90.8%) and ampicillin (85%) was observed. Almost all of the bacterial isolates were 100% sensitive to gentamycin and ciprofloxacin when used alone, and they were less resistant to cefotaxime (9.1%), vancomycin (6.8%), tetracycline (6.8%), kanamycin (4.5%) streptomycin (3.4%) and tobramycin (1.1%) (**Table 4**). Drug resistance and the sensitivity pattern of the isolates were determined by the Kirby-Bauer disk diffusion method using the CLSI 2014 guidelines. A multi-drug resistant nature was found in 80 (91.9%) of the isolates. The data in Table 4 clearly demonstrate that 86.1% of gram-negative bacterial isolates exhibited multi-drug resistance to at least 5–8 different antibiotics. Furthermore, 100% of the *S*. *aureus* isolates were resistant to three antibiotics that were employed for antibiotic susceptibility test.

### Isolation of bacteriophages and lytic efficacy in *in vitro* conditions

**Fig 3:**
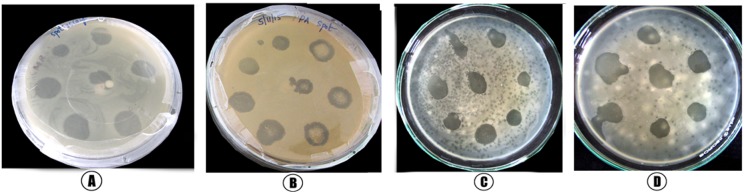
Plaque formation of lytic phages on double layer agar plates. Plaque assay of lytic phages on a lawn of MDR-bacterial isolates. A. Plaque assay of phage MDR-SA1 on the lawn of MDR-*S*. *aureus*. B. Plaque assay of phage MDR-PA4 on the lawn of MDR-*P*. *aeruginosa*. C. Plaque assay of phage MDR-KP1 on the lawn of MDR-*K*. *Pneumoniae*. D. Plaque assay of phage MDR-EC3 on the lawn of MDR-*E*. *Coli*.

The specificity shown by phages isolated against each of the MDR-bacteria included in this study was tested by plaque formation on the double layer agar plate (**Fig 3**) The isolated phage specific for *E*. *coli* (EC DP3) formed multiple small and irregular elongated plaques (**Fig 3A**); the phage PA DP4 formed numerous small and circular shaped plaques on the DLA plate (**Fig 3B**); the phage specific for *K*. *pneumoniae* (KP DP1) demonstrated plaque sizes ranging from small to large with circular shapes (**Fig 3C**); and the *S*. *aureus* phage, that is, SA DP1, formed well demarcated isolated big plaques on the top agar plate (**Fig 3D**). The titers of phages were determined using log dilutions of the purified phage lysate. These phages were named PA DP4 (*P*. *aeruginosa*), KP DP1 (*K*. *pneumoniae*), SA DP1(*S*. *aureus*) and EC DP3 (*E*. *coli*), corresponding to the host, and the titers were 3.72 x10^6^ PFU^-1^, 1.82 x 10^6^ PFU^-1^, 2.6 x 10^−7^ PFU^-1^ and 4.2 x10^7^ PFU^-1^, respectively.

**Fig 4:**
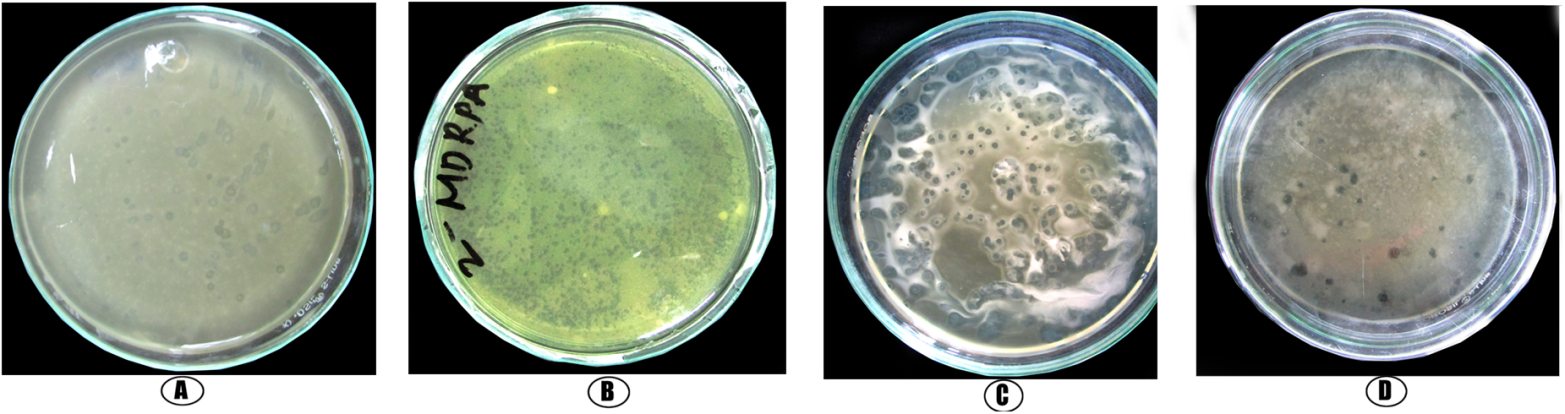
Spot assay of lytic phages on the MDR-bacteria from septic wounds. **Spot assay of lytic phages on the lawn of MDR-bacterial isolates**. A. Spot assay of phage MDR-SA1 on the lawn of multi-drug resistant *S*. *aureus*. B. Spot assay of phage MDR-PA4 on the lawn of multi-drug resistant *P*. *aeruginosa*. C. Spot assay of phage MDR-KP1 on the lawn of multi-drug resistant *K*. *pneumoniae*. D. Spot assay of phage MDR-EC3 on the lawn of multi-drug resistant *E*. *coli*.

The spot assay method was employed to further characterize the host specificity. SA DP1 formed irregular, dense spots (Fig 4A). The PA DP4 phage formed larger spots and had a core area in the lysed zones featuring dense bacterial lawns with clear edges (Fig 4B), whereas the *E*. *coli* phage formed clear large zones along with small plaques scattered throughout the top agar plate (Fig 4C). In the case of the KP DP1 phage, cleared inhibition zones with limited number of small plaques on the agar plate (Fig 4D) were observed.

**Fig 5:**
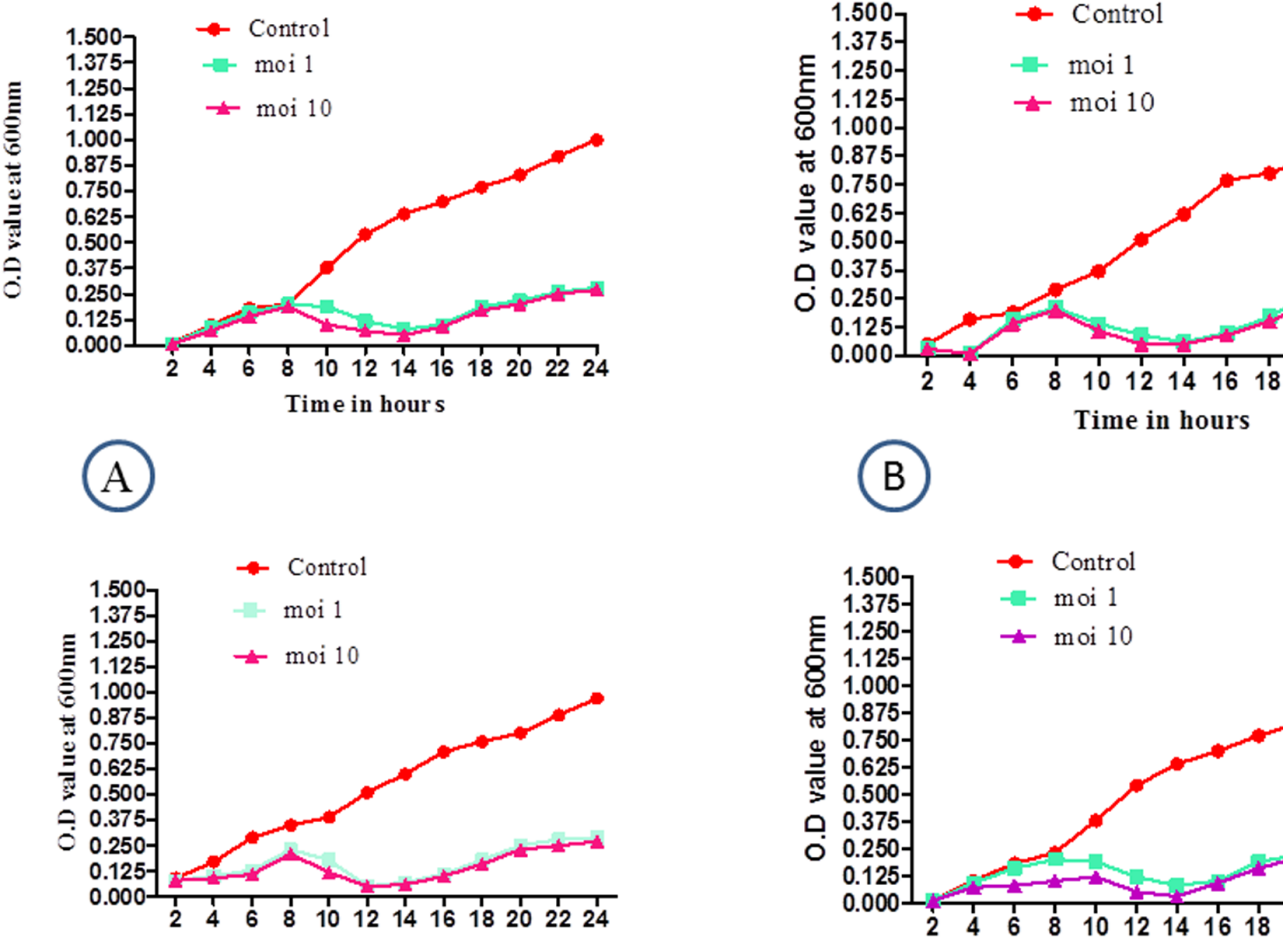
Effect of bacteriophages on the respective bacteria *in vitro*. Reduction of bacterial growth by corresponding phages compared with control. A. MDR-KP1 (control), phage KP DP1 at m.o.i 1 and 10 (test). B. MDR-SA1 (control), phage SA DP1 at m.o.i 1.

The isolated phages were further tested against MDR-bacterial isolates from septic wounds using a bacterial reduction assay in liquid medium to ascertain the lytic function. *In vitro* lysis of MDR-bacteria by the corresponding bacteriophages was monitored for 24 h during incubation at 37°C at 120 x g on an orbital shaker. The bacterial reductions assay by phages were compared with the corresponding controls. Phage infections produced a drastic decrease in MDR-bacterial optical density values at m.o.i 1 and 10 compared to the corresponding controls (**Fig 5**). The phages demonstrated maximum lytic activity at 12–14 h after incubation. KP DP1 (**Fig 5A**) demonstrated the minimum absorbance at 14 h, that is, 0.05 and 0.08 at m.o.i 10 and m.o.i 1, respectively, whereas the control flask showed 0.64 absorbance, indicating normal bacterial growth kinetics. In the case of phage SA DP1 (**Fig 5B**), the minimum OD at 12 h of incubation at m.o.i 10 was 0.05 and 0.09 at m.o.i 1, whereas the control flask demonstrated an absorbance of 0.51. The phage EC DP3 (**Fig 5C**) demonstrated a minimum OD at 14 h and then ODs of 0.03 and 0.08 at m.o.i 10 and 1, respectively, while the control demonstrated an OD of 0.64. The phage specific for *P*. *aeruginosa* (**Fig 5D**) demonstrated a minimum OD at 12 h of incubation (0.05), and the OD for both m.o.i 1 and 10 and the control flask were demonstrated to be 0.51. We noticed a reduction in bacterial population densities beginning at 4 h, which attained a peak reduction between 12–14 h for all four different phages tested in this study. However, constant increases in bacterial densities were observed at OD 600, after 14–16 h of incubation time, which was attributed to the growth of phage resistant bacteria or the inactivation of phages after that time point.

## Discussion

Skin provides an innate immune barrier and controls microbial colonization on the skin surface, protecting the underlying tissue from potential pathogens. The destruction of skin integrity leads to wounds. The microorganisms that infect wounds and damaged skin depend on the microbes present in the environment, the person’s immune system, and the depth of the wound. The risk of septic wounds is well known, and it is one of the major causes of hospitalization in patients suffering from external injuries (wounds) and diabetic patients. According to Tayfour, 10–33% of septic wounds were observed in India [34], and wound infections were primarily caused by different microbes, such as *S*. *aureus*, *Non-coagulase Streptococcus*, *Enterococci*, *E*.*coli*, *P*. *aeruginosa*, *K*. *pneumoniae*, *Enterobacter*, *Streptococci*, *Candida and Acinetobacter* [35].

The current study revealed that the rate of wound infection was more common in males (69.2%) than in females (30.7%). This is in agreement with research performed in different parts of India [36] and other countries [37]. Males are involved in occupations such as agriculture, industrial work, and construction work involving professional hazards where wounds are more likely to occur.

We noticed that 78.4% of pus samples demonstrated bacterial growth in selective medium and agar medium; this rate of isolation of pathogens is relatively higher than those in previous reports, 70.5% in [38] and 55.5% in [39]. Out of the total bacterial isolates, 61% were gram-negative bacteria, and 39% were gram-positive bacteria. In a similar type of study conducted in [40], gram-negative bacteria were predominantly found. Most clinical reports demonstrated that gram-negative bacteria are more predominant than gram-positive bacteria in most hospital-based environments [41].

This study revealed that 93% of culture positive wounds demonstrated mono-microbial growth, and 7% demonstrated poly-microbial growth. Similarly, a high percentage of mono-microbial growth has been reported in India and other countries (86–100% in India, 98% in Pakistan, 91.6% in southwest Ethiopia, and 53% in Nepal), [42], but these results contradict reports from Brazil [43].

Among these isolated pathogens, *P*. *aeruginosa* occurred the most predominantly in total isolates (22.6%), as previously reported (22%) [44], while *S*. *aureus* was the second most predominant isolate (19.1%). Our observations demonstrate a relatively lower percentage of prevalence than previous reports (*P*. *aeruginosa* (29.9%) and *S*. *aureus* (27.5%) by Thanni [45]; compared to the results of [40], *P*. *aeruginosa* (19%) was relatively higher in our isolates. Sankaran noted that *E*. *coli* was the predominant isolate, but this observation contradicts our findings, where we report *P*. *aeruginosa* as the predominant bacterium ([46] *Klebsiella* spp. (19.4%), [47] *S*. *aureus*, and [48] *Escherichia coli*). Generally, the rate of *P*. *aeruginosa* is high in wound infections, particularly in developing countries, because of the lack of high quality antiseptics and medicinal solutions and the lack of good hygienic conditions [36].

The average resistance of the selected antibiotics was very high in both gram-positive and gram-negative isolates, up to approximately 91.1%. Interestingly, the study conducted by Mulu [49] in Ethiopia demonstrated a relatively higher percentage of multi-drug resistance in comparison to our observations (95.5 to 100%). High multi-drug resistance in the isolates may be due to self-medication by patients, the lack of diagnostic laboratory services or the unavailability of guidelines about the selection of drugs, thereby leading to the inappropriate use of antibiotics. *S*. *aureus* was 100% resistant to benzyl penicillin, ampicillin, and amoxicillin and had variable resistances to other tested drugs, including kanamycin (13.6%), vancomycin (9%), cefotaxime (9%) and tobramycin (4.7%). The rate of antibiotic resistance observed was consistent with another study from India [36]. Further, only 9% of *S*. *aureus* were resistant to vancomycin, as noted in this study, which was much lower than the 40% reported in Iran by Mirnejad [50]. The MDR isolates from the septic wounds of patients were extremely sensitive to gentamycin, ciprofloxacin, streptomycin and tetracycline.

In this study, *P*. *aeruginosa* demonstrated resistance to benzyl penicillin (92.3%), amoxicillin (88.4%), ampicillin (80.7%), cefotaxime (38.5%), kanamycin, tetracycline and vancomycin (11.3%). The antibiotic resistance pattern of the *P*. *aeruginosa* isolates was relatively higher than those found in previous reports [51]. Our study notes that *K*. *pneumoniae* demonstrated resistance to benzyl penicillin and amoxicillin (85% each), ampicillin (75%), cefotaxime (20%), kanamycin (10%) and vancomycin (5%), while these isolates were highly sensitive to kanamycin, tobramycin, streptomycin, gentamycin, tetracycline, and ciprofloxacin. Our results are positively correlated with previous studies conducted in India by Goswami [36]. In our study, we report resistant pattern of *E*. *coli* isolates to benzyl penicillin (89.4%), amoxicillin and ampicillin (84.2%), and tetracycline (57.8%); however, streptomycin demonstrated only 42.1%. The sensitivity patterns exhibited by *E*. *coli* were relatively lower than those in prior reports [48], which demonstrated (ampicillin (96.6%), tetracycline (79%) and gentamicin (51.7%)) a relatively higher percentage of drug resistance than our findings in the current study.

We note that the most effective antibiotic drugs against septic wounds caused by MDR-bacterial isolates are gentamycin and ciprofloxacin. Another research group from Nigeria reported that gentamycin is an effective drug for MDR-bacteria in septic wounds [6]. Due to the alarming level of MDR-bacterial evolution, alternative therapeutics are required for sepsis causing MDR-bacteria and for other diseases where MDR-bacteria are prevalent. Most importantly, bacteriophages can be tested as reliable alternatives to antibiotics. In this study, we standardized isolation and *in vitro* lytic activity of bacteriophages against MDR-bacteria infecting the septic wounds.

We determined the host specificity of bacteriophages using the soft agar method, spot assay methods and *in vitro* lytic efficacy of bacteriophages on MDR-bacteria from septic wounds were examined by a bacterial reduction assay. Our results agree with several other reports, conclusively demonstrating that bacteriophages function as therapeutic agents against bacterial infections. Bacteriophages have been employed to cure infectious diseases caused by both gram-positive and gram-negative bacteria, such as *E*. *coli*, *P*. *aeruginosa*, *A*. *baumannii*, *K*. *pneumoniae*, *V*. *vulnificus*, *Salmonella* spp., *S*. *pyogenes*, *E*. *faecium* and *S*. *aureus* [29–31,49–51]. Literatures from past studies demonstrate that compared to chemotherapy, phage therapy demonstrates positive results and seems to be a reliable agent to replace antibiotics in the future [52,53].

These isolated and positively tested phages (PA DP4, KP DP1, SA DP1, and EC DP3) were rescreened for lytic activity on the MDR-bacteria from septic wounds by a bacterial reduction assay. *In vitro* results demonstrate that MDR-bacterial isolates could regrow after 14–16 h of phage therapy; this may be due to emergence of phage resistant strains, as also reported by the Vieira, Kumari, and Andretti groups [54,55].

At present, antibiotic drugs, such as ciprofloxacin and gentamicin, are the most effective regimen to treat MDR-bacteria, such as *P*. *aeruginosa*, *S*. *aureus*, *K*. *pneumoniae* and *E*. *coli*, involved in septic wound infections. Due to the evolution of resistance to these commonly used drugs, there is now a need for alternative agents to treat MDR-bacteria. Bacteriophages can lyse MDR-bacterial isolates that cause septic wounds. Bacteriophages have bactericidal activity against the pathogenic bacteria responsible for diseases, and they may quickly reduce bacterial loads. The use of bacteriophages has been reported as an attractive method for treating *E*. *coli*, *P*. *aeruginosa*, *K*. *pneumoniae* and *S*. *aureus* infections [55] in various disease conditions. Limitations of the present study are following:

1. Immune system is expected to mount response against phages and may eliminate phages from circulation by producing antibodies and cell mediated responses.
2. Endotoxins of bacterium may contaminate the phage preparations leading to undesired responses.
3. Extensive study is needed to understand the basic science of phage and human interactions in short term and long term usage of phage for theraupatic purpose either in single preparations or as phage cocktail **[56].**

From our results, we demonstrated that both MDR-gram-positive bacteria (*S*. *aureus*) and gram-negative bacteria (*P*. *aeruginosa*, *E*.*coli* and *K*. *pneumoniae*) obtained from septic wounds were susceptible to bacteriophage lysis (PA DP4, SA DP1, KP DP1, and EC DP3), and these phages deserve further study for optimization under *in vivo* conditions using the appropriate mouse model.

## Conclusion

The most common bacteria isolated from septic wound infections were *P*. *aeruginosa*, followed by *S*. *aureus*, *K*. *pneumoniae* and *E*. *coli*. These isolates had a high frequency of resistance to ampicillin, benzylpenicillin, amoxicillin, vancomycin and tetracycline. The bacteriophages isolated from sewage demonstrated lytic efficacy against MDR-bacterial isolates from septic wounds in an *in vitro* assay. These phages, which exhibited lytic activity and reduced the bacterial load, may be viewed as alternative agents to antibiotics. In the future, phage therapy will be a reliable way to treat MDR-bacterial infections, although much work is needed to understand all the mechanisms involved, including use of phage cocktails for multiple bacterial infections.

## Supporting information

S1 Fig Growth of MDR-bacterial isolates on nutrient and selective media. A. Growth of *Staphylococcus aureus* on Nutrient agar plate, B. Growth of *Staphylococcus aureus* on mannitol salt agar plate, C. Growth of *Klebsiella pnemoniae* on Nutrient agar plate, D. Growth of *Klebsiella pnemoniae* on Mac Conkey agar plate, E. Growth of *Escherichia coli* on Nutrient agar plate, F. Growth of *Escherichia coli* on Mac Conkey agar plate, G. Growth of *Pseudomonas aeruginosa* on Nutrient agar plate, H. Growth of *Pseudomonas aeruginosa* on Cetrimide agar plate.
(TIF)

S2 Fig Pus swab sample collections from the septic wound patients admitted in the RIMS hospital. (DOCX)

S1 Data Raw data of septic wound patients, sex and age factors. Raw data of patients admitted in RIMS hospital, Kadapa, India from 2014–2015. Bacterial Positive samples with wounds types, age groups with sex ratio were included in this study. We uploaded the total 130 septic wound suspected patients in this study.
(DOCX)

S1 Table Statistics of Table 1, Table 2 and Table 3. (DOCX)

## References

[1] Ndip RN, Takang A, Echakachi CM, Malongue A, Akoachere J (2007) In-vitro antimicrobial activity of selected honeys on clinical isolates of Helicobacter pylori. African health sciences 7. PMC3074369 21499488

[2] Dai T, Huang Y-Y, K Sharma S, T Hashmi J, B Kurup D (2010) Topical antimicrobials for burn wound infections. Recent patents on anti-infective drug discovery 5: 124–151. 20429870 10.2174/157489110791233522 PMC2935806

[3] Church D, Elsayed S, Reid O, Winston B, Lindsay R (2006) Burn wound infections. Clinical microbiology reviews 19: 403–434. doi: 10.1128/CMR.19.2.403-434.2006 16614255 10.1128/CMR.19.2.403-434.2006 PMC1471990

[4] Bisno AL, Stevens DL (1996) Streptococcal infections of skin and soft tissues. New England Journal of Medicine 334: 240–246. doi: 10.1056/NEJM199601253340407 8532002 10.1056/NEJM199601253340407

[5] Janda J, Abbott S, Brenden R (1997) Overview of the etiology of wound infections with particular emphasis on community-acquired illnesses. European journal of clinical microbiology & infectious diseases 16: 189–201. 9131321 10.1007/BF01709581

[6] Anguzu J, Olila D (2007) Drug sensitivity patterns of bacterial isolates from septic post-operative wounds in a regional referral hospital in Uganda. African health sciences 7. 10.5555/afhs.2007.7.3.148 PMC2269712 18052868

[7] Mehta M, Dutta P, Gupta V (2007) Bacterial isolates from burn wound infections and their antibiograms: A eight-year study. Indian Journal of plastic surgery 40: 25.

[8] Kotz P, Fisher J, McCluskey P, Hartwell SD, Dharma H (2009) Use of a new silver barrier dressing, ALLEVYN? Ag in exuding chronic wounds. International wound journal 6: 186–194. doi: 10.1111/j.1742-481X.2009.00608.x 19538192 10.1111/j.1742-481X.2009.00608.x PMC2737751

[9] Amoran O, Sogebi A, Fatugase O (2013) Rates and Risk Factors Associated with Surgical Site Infections in a Tertiary Care Center in South-Western Nigeria.

[10] Sheridan RL (2005) Sepsis in pediatric burn patients. Pediatric Critical Care Medicine 6: S112–S119. doi: 10.1097/01.PCC.0000161577.27849.BE 15857543 10.1097/01.PCC.0000161577.27849.BE

[11] Hart C, Kariuki S (1998) Antimicrobial resistance in developing countries. British medical journal 317: 647 9727995 10.1136/bmj.317.7159.647 PMC1113834

[12] Esebelahie N, Newton-Esebelahie F, Omoregie R (2013) Aerobic bacterial isolates from infected wounds. African Journal of Clinical and Experimental Microbiology 14: 155–159.

[13] Shittu A, Kolawole D, Oyedepo E (2002) A study of wound infections in two health institutions in Ile-Ife, Nigeria. African journal of biomedical research 5. 12732758

[14] Shahi SK, Kumar A (2015) Isolation and Genetic Analysis of Multidrug Resistant Bacteria from Diabetic Foot Ulcers. Frontiers in microbiology 6. 10.3389/fmicb.2015.01464 PMC4700134 26779134

[15] Buteera A, Byimana J (2009) Principles of management of open fractures.

[16] Nkang O, Okonko I, Mejeha O, Adewale O, Udeze A (2009) Assessment of antibiotics susceptibility profiles of some selected clinical isolates from laboratories in Nigeria. Journal of Microbiology and Antimicrobials 1: 019–026.

[17] Kutateladze M, Adamia R (2010) Bacteriophages as potential new therapeutics to replace or supplement antibiotics. Trends in biotechnology 28: 591–595. doi: 10.1016/j.tibtech.2010.08.001 20810181 10.1016/j.tibtech.2010.08.001

[18] Organization WH (2014) Antimicrobial resistance global report on surveillance: 2014 summary.

[19] Golkar Z, Bagasra O, Jamil N (2013) Experimental phage therapy on multiple drug resistant Pseudomonas aeruginosa infection in mice. Journal of Antivirals & Antiretrovirals 2013.

[20] Hall AR, De Vos D, Friman V-P, Pirnay J-P, Buckling A (2012) Effects of sequential and simultaneous applications of bacteriophages on populations of Pseudomonas aeruginosa in vitro and in wax moth larvae. Applied and environmental microbiology 78: 5646–5652. doi: 10.1128/AEM.00757-12 22660719 10.1128/AEM.00757-12 PMC3406105

[21] Chan BK, Abedon ST, Loc-Carrillo C (2013) Phage cocktails and the future of phage therapy. Future microbiology 8: 769–783. doi: 10.2217/fmb.13.47 23701332 10.2217/fmb.13.47

[22] Haq IU, Chaudhry WN, Akhtar MN, Andleeb S, Qadri I (2012) Bacteriophages and their implications on future biotechnology: a review. Virology journal 9: 1. 22234269 10.1186/1743-422X-9-9 PMC3398332

[23] Wang Z, Zheng P, Ji W, Fu Q, Wang H (2016) SLPW: A Virulent Bacteriophage Targeting Methicillin-Resistant Staphylococcus aureus In Vitro and In Vivo. Frontiers in microbiology 7: 934 doi: 10.3389/fmicb.2016.00934 27379064 10.3389/fmicb.2016.00934 PMC4908117

[24] Hudzicki J (2009) Kirby-Bauer disk diffusion susceptibility test protocol. American Society for Microbiol.

[25] Hsueh P-R, Ko W-C, Wu J-J, Lu J-J, Wang F-D (2010) Consensus statement on the adherence to Clinical and Laboratory Standards Institute (CLSI) Antimicrobial Susceptibility Testing Guidelines (CLSI-2010 and CLSI-2010-update) for Enterobacteriaceae in clinical microbiology laboratories in Taiwan. Journal of Microbiology, Immunology and Infection 43: 452–455. 10.1016/S1684-1182(10)60070-9 21075714

[26] Smith HW, Huggins MB, Shaw KM (1987) The control of experimental Escherichia coli diarrhoea in calves by means of bacteriophages. Microbiology 133: 1111–1126. 10.1099/00221287-133-5-1111 3309177

[27] Cerveny KE, DePaola A, Duckworth DH, Gulig PA (2002) Phage therapy of local and systemic disease caused by Vibrio vulnificus in iron-dextran-treated mice. Infection and immunity 70: 6251–6262. doi: 10.1128/IAI.70.11.6251-6262.2002 12379704 10.1128/IAI.70.11.6251-6262.2002 PMC130292

[28] Bourdin G, Schmitt B, Guy LM, Germond J-E, Zuber S (2014) Amplification and purification of T4-like Escherichia coli phages for phage therapy: from laboratory to pilot scale. Applied and environmental microbiology 80: 1469–1476. doi: 10.1128/AEM.03357-13 24362424 10.1128/AEM.03357-13 PMC3911048

[29] Ceyssens P-J, Lavigne R, Mattheus W, Chibeu A, Hertveldt K (2006) Genomic analysis of Pseudomonas aeruginosa phages LKD16 and LKA1: Establishment of the φKMV subgroup within the T7 supergroup. Journal of bacteriology 188: 6924–6931. doi: 10.1128/JB.00831-06 16980495 10.1128/JB.00831-06 PMC1595506

[30] Santos SB, Carvalho CM, Sillankorva S, Nicolau A, Ferreira EC (2009) The use of antibiotics to improve phage detection and enumeration by the double-layer agar technique. BMC microbiology 9: 1. 19627589 10.1186/1471-2180-9-148 PMC2728735

[31] Armon R, Kott Y (1993) A simple, rapid and sensitive presence/absence detection test for bacteriophage in drinking water. Journal of applied bacteriology 74: 490–496. 8486556 10.1111/j.1365-2672.1993.tb05159.x

[32] Sambrook J, Russell DW (2001) Molecular cloning: a laboratory manual 3rd edition Coldspring-Harbour Laboratory Press, UK.

[33] Edwards-Jones V, Greenwood JE, Group MBR (2003) What’s new in burn microbiology?: James Laing memorial prize essay 2000. Burns 29: 15–24. 12543040 10.1016/s0305-4179(02)00203-6

[34] Wilson A, Gibbons C, Reeves B, Hodgson B, Liu M (2004) Surgical wound infection as a performance indicator: agreement of common definitions of wound infection in 4773 patients. Bmj 329: 720 doi: 10.1136/bmj.38232.646227.DE 15367425 10.1136/bmj.38232.646227.DE PMC518898

[35] Tayfour MA, Al-Ghamdi SM, Al-Ghamdi AS (2005) Surgical wound infections in King Fahad Hospital at Al-Baha. Saudi medical journal 26: 1305–1306. 16127538

[36] Goswami NN, Trivedi HR, Goswami APP, Patel TK, Tripathi C (2011) Antibiotic sensitivity profile of bacterial pathogens in postoperative wound infections at a tertiary care hospital in Gujarat, India. Journal of Pharmacology and pharmacotherapeutics 2: 158 doi: 10.4103/0976-500X.83279 21897707 10.4103/0976-500X.83279 PMC3157123

[37] Ohalete C, Obi R, EmeaKoroha M (2012) Bacteriology of different wound infection and their antimicrobial susceptibility patterns in Imo state Nigeria. World J Pharm Pharm Sci 1: 1155–1172.

[38] Azene MK, Beyene BA (2011) Bacteriology and antibiogram of pathogens from wound infections at Dessie Laboratory, North East Ethiopia. Tanzania journal of health research 13. 10.4314/thrb.v13i4.64901 26592050

[39] Kaftandzieva A, Cekovska Z, Kaftandziev I, Petrovska M, Panovski N (2012) Bacteriology of Wound-Clinical Utility of Gram Stain Microscopy and the Correlation with Culture. Macedonian Journal of Medical Sciences 5: 72–77.

[40] Iregbu K, Uwaezuoke N, Nwajiobi-Princewill I, Eze S, Medugu N, et al A profile of wound infections in National Hospital Abuja. African Journal of Clinical and Experimental Microbiology 14: 160–163.

[41] Mehta PA, Cunningham CK, Colella CB, Alferis G, Weiner LB (2000) Risk factors for sternal wound and other infections in pediatric cardiac surgery patients. The Pediatric infectious disease journal 19: 1000–1004. 11055604 10.1097/00006454-200010000-00012

[42] Hout B, Oum C, Men P, Vanny V, Supaprom C (2015) Drug resistance in bacteria isolated from patients presenting with wounds at a non-profit Surgical Center in Phnom Penh, Cambodia from 2011–2013. Tropical Diseases, Travel Medicine and Vaccines 1: 1. 10.1186/s40794-015-0006-5 PMC5526368 28883936

[43] Perim MC, Borges JdC, Celeste SRC, Orsolin EdF, Mendes RR (2015) Aerobic bacterial profile and antibiotic resistance in patients with diabetic foot infections. Revista da Sociedade Brasileira de Medicina Tropical 48: 546–554. doi: 10.1590/0037-8682-0146-2015 26516963 10.1590/0037-8682-0146-2015

[44] Sikka R, Mann J, Deep VM, Chaudhary U, Deep A (2012) Prevalence and antibiotic sensitivity pattern of bacteria isolated from nosocomial infections in a surgical ward. Indian J Clin Pract 22: 519–525.

[45] Thanni LO, Osinupebi OA, Deji-Agboola M (2003) Prevalence of bacterial pathogens in infected wounds in a tertiary hospital, 1995–2001: any change in trend? Journal of the National Medical Association 95: 1189 14717475 PMC2594861

[46] Sankaran SV, Rajagopal GK, Achamkulangara S (2016) Effects of antibiotic prophylaxis on surgical wounds: A study in a tertiary care centre of central Kerala. Journal of The Academy of Clinical Microbiologists 18: 12.

[47] Ayub M, Rizwan H, Siddique S, Maryam U (2015) Isolation of Pathogens Causing Sepsis, Pus and Infected Wounds from Critical Care Unit: A Retrospective Study. Annals of Clinical and Laboratory Research.

[48] Mama M, Abdissa A, Sewunet T (2014) Antimicrobial susceptibility pattern of bacterial isolates from wound infection and their sensitivity to alternative topical agents at Jimma University Specialized Hospital, South-West Ethiopia. Annals of clinical microbiology and antimicrobials 13: 1. 24731394 10.1186/1476-0711-13-14 PMC4017222

[49] Mulu W, Kibru G, Beyene G, Damtie M (2012) Postoperative nosocomial infections and antimicrobial resistance pattern of bacteria isolates among patients admitted at Felege Hiwot Referral Hospital, Bahirdar, Ethiopia. Ethiopian journal of health sciences 22: 7–18. PMC3437975 22984327

[50] Mirnejad R, Fallahi S, Kiani J, Jeddi F, Khoobdel M (2008) Epidemic assessment of bacterial agents in osteomyelitis and their antibiotic resistance pattern determination. Journal of Biological Sciences 8: 478–481.

[51] Upadhyay A, Maharjan R, Shakya B (2012) Multidrug resistance bacteria in different clinical samples in National Medical College and Teaching Hospital Birgunj, Nepal. Res J Pharm, BiolChemSci (RJPBCS) 3: 797–707.

[52] Mathur M, Vidhani S, Mehndiratta P, Bhalla P, Reddy B (2003) Bacteriophage therapy: an alternative to conventional antibiotics. JOURNAL-ASSOCIATION OF PHYSICIANS OF INDIA 51: 593–596. 15266928

[53] Nouraldin AAM, Baddour MM, Harfoush RAH, Essa SAM (2016) Bacteriophage-antibiotic synergism to control planktonic and biofilm producing clinical isolates of Pseudomonas aeruginosa. Alexandria Journal of Medicine 52: 99–105.

[54] Kumari S, Harjai K, Chhibber S (2010) Evidence to support the therapeutic potential of bacteriophage Kpn5 in burn wound infection caused by Klebsiella pneumoniae in BALB/c mice. J Microbiol Biotechnol 20: 935–941. 20519918 10.4014/jmb.0909.09010

[55] Vieira A, Silva Y, Cunha A, Gomes N, Ackermann H-W (2012) Phage therapy to control multidrug-resistant Pseudomonas aeruginosa skin infections: in vitro and ex vivo experiments. European journal of clinical microbiology & infectious diseases 31: 3241–3249. 22777594 10.1007/s10096-012-1691-x

[56] Sulakvelidze A, Alavidze Z, Morris JG (2001) Bacteriophage therapy. Antimicrobial agents and chemotherapy 45: 649–659. doi: 10.1128/AAC.45.3.649-659.2001 11181338 10.1128/AAC.45.3.649-659.2001 PMC90351

