# Supplementary figures and images for: Isolation and in vitro evaluation of bacteriophages against MDR-bacterial isolates from septic wound infections
Source: PLoS One. 2017 Jul 18;12(7):e0179245. doi: 10.1371/journal.pone.0179245 (PMC5515400; doi:10.1371/journal.pone.0179245)

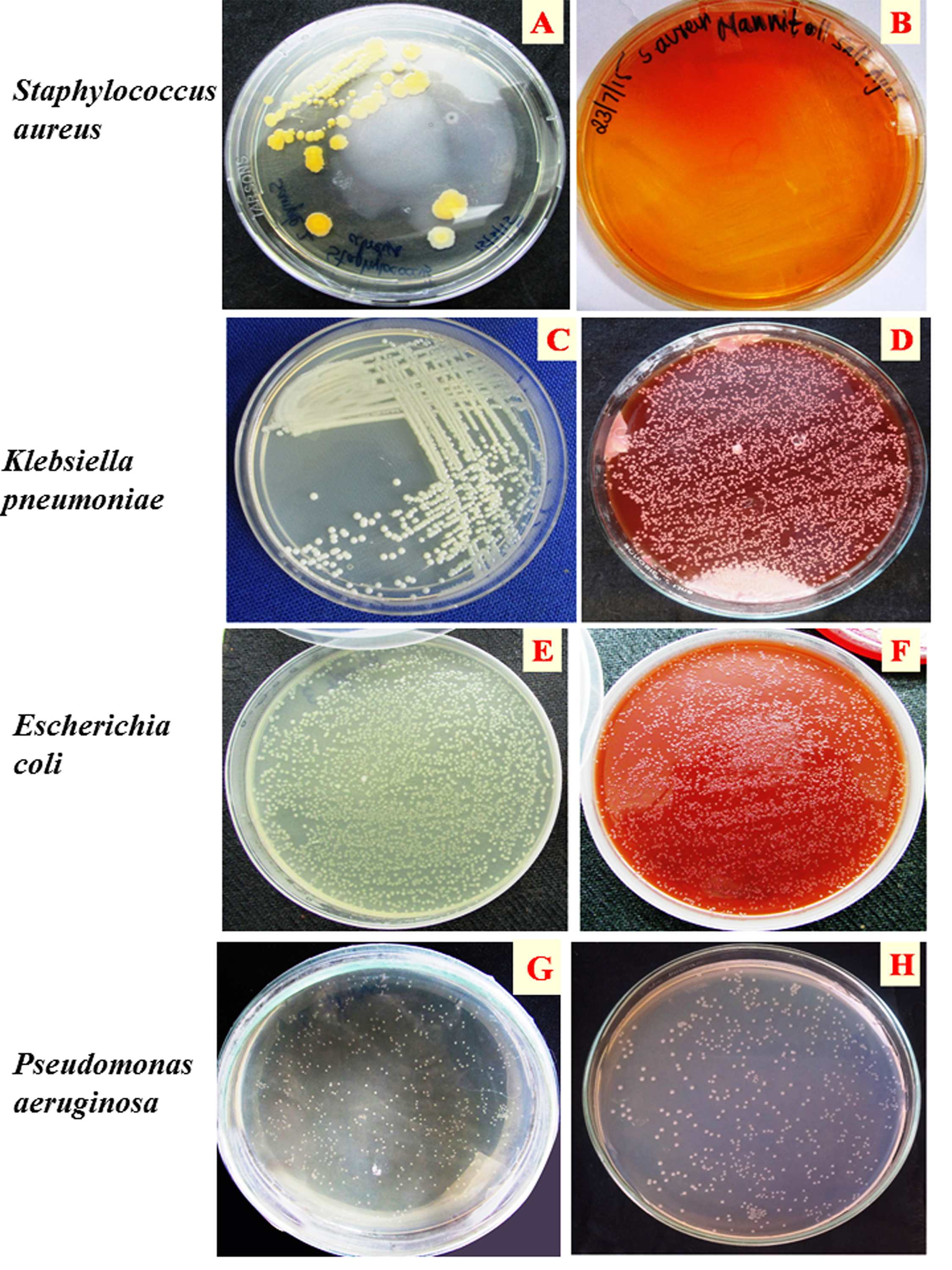

Supplement: S1 Fig — A. Growth of Staphylococcus aureus on Nutrient agar plate, B. Growth of Staphylococcus aureus on mannitol salt agar plate, C. Growth of Klebsiella pnemoniae on Nutrient agar plate, D. Growth of Klebsiella pnemoniae on Mac Conkey agar plate, E. Growth of Escherichia coli on Nutrient agar plate, F. Growth of Escherichia coli on Mac Conkey agar plate, G. Growth of Pseudomonas aeruginosa on Nutrient agar plate, H. Growth of Pseudomonas aeruginosa on Cetrimide agar plate. (TIF) [file pone.0179245.s001.tif]

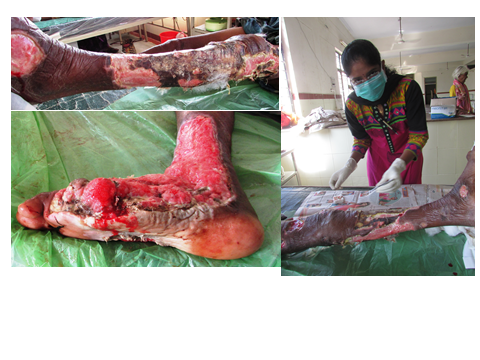

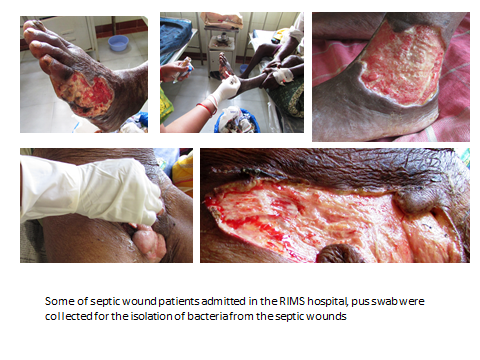

Supplement: S2 Fig — (DOCX) [file pone.0179245.s002.docx]
