# Supplementary material for: Isolation and in vitro evaluation of bacteriophages against MDR-bacterial isolates from septic wound infections
Source: PLoS One. 2017 Jul 18;12(7):e0179245. doi: 10.1371/journal.pone.0179245 (PMC5515400; doi:10.1371/journal.pone.0179245)
Supplement: S1 Data — Raw data of patients admitted in RIMS hospital, Kadapa, India from 2014–2015. Bacterial Positive samples with wounds types, age groups with sex ratio were included in this study. We uploaded the total 130 septic wound suspected patients in this study. (DOCX) [file pone.0179245.s003.docx]

| No.of patients | WOUND TYPE | | AGE | SEX |
| --- | --- | --- | --- | --- |
| 1. | Diabetic wounds | | 59 | M |
| 2. | // | | 58 | M |
| 3. | // | | 51 | M |
| 4. | // | | 55 | F |
| 5. | // | | 59 | M |
| 6. | // | | 49 | M |
| 7. | // | | 47 | M |
| 8. | Burn wounds | | 9 | F |
| 9. | // | | 31 | F |
| 10. | // | | 11 | M |
| 11. | // | | 41 | F |
| 12. | Diabetic wounds | | 59 | M |
| 13. | // | | 39 | M |
| 14. | // | | 31 | M |
| 15. | // | | 49 | F |
| 16. | // | | 45 | F |
| 17. | // | | 53 | M |
| 18. | // | | 35 | M |
| 19. | // | | 49 | M |
| 20. | // | | 59 | F |
| 21 | // | | 16 | M |
| 22 | Post-operative wounds | | 18 | M |
| 23 | // | | 19 | M |
| 24 | // | | 55 | M |
| 25 | // | | 7 | M |
| 26 | // | | 28 | M |
| 27 | // | | 29 | M |
| 28 | // | | 59 | M |
| 29 | // | | 26 | M |
| 30 | // | | 22 | M |
| 31 | // | | 45 | M |
| 32 | // | | 43 | M |
| 33 | // | | 40 | F |
| 34 | Burn wounds | | 37 | F |
| 35 | // | | 39 | F |
| 36 | // | | 48 | F |
| 37 | // | | 44 | M |
| 38 | Diabetic wounds | | 45 | M |
| 39 | // | | 39 | M |
| 40 | // | | 45 | M |
| 41 | // | | 46 | M |
| 42 | // | | 48 | M |
| 43 | // | | 49 | F |
| 44 | // | | 50 | M |
| 45 | // | | 55 | F |
| 46 | // | | 60 | M |
| 47 | // | | 57 | M |
| 48 | // | | 38 | M |
| 49 | // | | 65 | M |
| 50 | // | | 63 | M |
| 51 | // | | 36 | M |
| 52 | // | | 69 | M |
| 53 | // | | 73 | M |
| 54 | Post- operative wounds | | 73 | F |
| 55 | // | 72 | | M |
| 56 | // | 70 | | M |
| 57 | // | 69 | | M |
| 58 | // | 65 | | M |
| 59 | // | 39 | | M |
| 60 | // | 70 | | M |
| 61 | // | 23 | | M |
| 62 | Burn wounds | 36 | | M |
| 63 | Post –operative wounds | 39 | | M |
| 64 | // | 44 | | M |
| 65 | Diabetic wounds | 75 | | F |
| 66 | // | 81 | | F |
| 67 | Burn wounds | 25 | | F |
| 68 | Diabetic wounds | 73 | | F |
| 69 | // | 58 | | F |
| 70 | // | 74 | | F |
| 71 | // | 38 | | M |
| 72 | Post-operative wounds | 37 | | M |
| 73 | // | 75 | | M |
| 74 | // | 49 | | M |
| 75 | Diabetic wounds | 48 | | M |
| 76 | // | 40 | | M |
| 77 | // | 55 | | M |
| 78 | // | 58 | | M |
| 79 | // | 59 | | M |
| 80 | // | 69 | | M |
| 81 | // | 36 | | M |
| 82 | // | 67 | | M |
| 83 | // | 56 | | M |
| 84 | Post-operative wounds | 82 | | M |
| 85 | // | 44 | | M |
| 86 | // | 23 | | M |
| 87 | // | 15 | | M |
| 88 | // | 11 | | M |
| 89 | // | 17 | | M |
| 90 | // | 23 | | M |
| 91 | // | 31 | | M |
| 92 | // | 11 | | M |
| 93 | // | 15 | | M |
| 94 | // | 17 | | M |
| 95 | // | 19 | | M |
| 96 | // | 28 | | M |
| 97 | Diabetic wound | 54 | | M |
| 98 | Burn wounds | 37 | | M |
| 99 | Post -operative wounds | 28 | | M |
| 100. | // | 60 | | M |
| 101. | // | 59 | | M |
| 102. | // | 72 | | M |
| 103. | // | 6 | | M |
| 104. | // | 29 | | F |
| 105. | // | 65 | | F |
| 106. | // | 73 | | M |
| 107. | // | 8 | | M |
| 108. | // | 71 | | M |
| 109. | // | 74 | | M |
| 110. | // | 83 | | M |
| 111. | // | 62 | | M |
| 112. | // | 60 | | M |
| 113. | // | 80 | | M |
| 114 | // | 79 | | M |
| 115. | // | 67 | | M |
| 116. | // | 54 | | M |
| 117. | // | 81 | | M |
| 118. | // | 56 | | M |
| 119. | // | 65 | | F |
| 120. | // | 61 | | M |
| 121. | // | 22 | | M |
| 122. | // | 63 | | M |
| 123. | // | 51 | | M |
| 124. | // | 59 | | M |
| 125. | // | 43 | | M |
| 126. | // | 49 | | M |
| 127. | // | 45 | | M |
| 128. | // | 50 | | M |
| 129. | // | 50 | | F |
| 130. | Burn wound | 21 | | F |

Raw data of patients admitted in RIMS hospital, Kadapa, India from 2014-2015. Bacterial Positive samples with wounds types, age groups with sex ratio were included in this study. We uploaded the total 102 bacterial positive samples subjects.
