# Supplementary material for: Isolation and in vitro evaluation of bacteriophages against MDR-bacterial isolates from septic wound infections
Source: PLoS One. 2017 Jul 18;12(7):e0179245. doi: 10.1371/journal.pone.0179245 (PMC5515400; doi:10.1371/journal.pone.0179245)
Supplement: S1 Table — (DOCX) [file pone.0179245.s004.docx]

| Table. 1 Analyzed by using GRAPH PAD PRISM SOFTWARE | Sex distribution of bacterial  isolates from septic wounds |
| --- | --- |
|  |  |
| Chi-square |  |
| Chi-square, degree of freedom | **1.505**, 4 |
| P value | **0.8258** |
| P value summary | ns |
| One- or two-sided | NA |
| Statistically significant? (alpha<0.05) | No |
|  |  |
| Data analyzed |  |
| Number of rows | 3 |
| Number of columns | 3 |

The overall statistics of the wound type with gender, age and type of bacterial is significantly not related to others, there is no effect on each other. This information was sorted from the raw data and by using GRAPH PAD PRISM SOFT WARE we get the statistics of the raw data. We reported all this information in below table. Please see the below table for the overall statistics of the raw data.

These results are copied from the output of graph pad prism software. We already informed this information in the manuscript.

| **Table.2 Analyzed by using GRAPH PAD PRISM SOFTWARE** | **Age groups with Significant bacterial growth** |  |  |
| --- | --- | --- | --- |
|  |  |  |  |
| One-way analysis of variance | |  |  |
| P value | **0.0485** |  |  |
| P value summary | * |  |  |
| Are means signif. different? (P < 0.05) | Yes |  |  |
| Number of groups | 3 |  |  |
| F | 3.597 |  |  |
| R square | 0.2856 |  |  |
|  |  |  |  |
| Bartlett's test for equal variances | |  |  |
| Bartlett's statistic (corrected) | 4.276 |  |  |
| P value | 0.1179 |  |  |
| P value summary | ns |  |  |
| Do the variances differ signif. (P < 0.05) | No |  |  |
|  |  |  |  |
| ANOVA Table | Sum of Squares | Degree of freedom | Mean of Squares |
| Treatment (between columns) | 1641 | 2 | 820.4 |
| Residual (within columns) | 4105 | 18 | 228.1 |
| Total | 5746 | 20 |  |

| **Table.3 Analysed by GRAPH PAD SOFT WARE** | **Wound type with bacterial type** |
| --- | --- |
| Column B | Gram positive |
| vs | vs |
| Column C | Gram negative |
|  |  |
| Unpaired t test |  |
| P value | 0.3072 |
| P value summary | ns |
| Are means signif. different? (P < 0.05) | No |
| One- or two-tailed P value? | Two-tailed |
| t-valve degree of freedom | t=0.7572 df=4 |
|  |  |
| How big is the difference? | |
| Mean ± SEM of column B | 15.00 ± 7.095 N=3 |
| Mean ± SEM of column C | 23.33 ± 8.413 N=3 |
| Difference between means | -8.333 ± 11.01 |
| 95% confidence interval | -38.88 to 22.22 |
| R square | 0.1254 |
|  |  |
| F test to compare variances | |
| F,DFn, Dfd | 1.406, 2, 2 |
| P value | 0.8312 |
| P value summary | ns |
| Are variances significantly different? | No |

Data analyzed: Two-way ANOVA with RM by columns

| Source of Variation | Degrees of Freedom | Sum of Squares | Mean square |
| --- | --- | --- | --- |
| wound type | 1.0 | 456.3 | 456.3 |
| gender | 2.0 | 418.2 | 209.1 |
| Type of bacteria | 2.0 | 131.2 | 65.58 |
| Residual (error) | 6.0 | 700.0 | 116.7 |
| Total | 11.0 | 1706 |  |

**Does wound type have the same effect at all values of gender?**

Interaction accounts for 7.69% of the total variance.

F = 0.56. DFn=2 DFd=6

The P value = 0.5974

**If there is no interaction overall, there is a 60% chance of randomly observing so much**

**interaction in an experiment of this size. The interaction is considered not significant.**

**Does wound type affect the result?**

wound type accounts for 26.75% of the total variance.

F = 3.91. DFn=1 DFd=6

The P value = 0.0953

**If wound type has no effect overall, there is a 9.5% chance of randomly observing an**

**effect this big (or bigger) in an experiment of this size. The effect is considered**

**not quite significant.**

**Does gender affect the result?**

gender accounts for 24.52% of the total variance.

F = 1.79. DFn=2 DFd=6

The P value = 0.2453

**If gender has no effect overall, there is a 25% chance of randomly observing an**

**this big (or bigger) in an experiment of this size. The effect is considered not significant.**
